# Precariousness Represents an Independent Risk Factor for Depression in Children With Sickle Cell Disease

**DOI:** 10.1155/2024/1689091

**Published:** 2024-10-24

**Authors:** Narcisse Elenga, Janaine Lony, Joddy Mafemamissindu, Noelis Thomas Boizan, Lindsay Osei, Mathieu Nacher

**Affiliations:** ^1^Sickle Cell Disease Center, Cayenne Medical Center, Rue des flamboyants, BP 6006, French Guiana, Cayenne Cedex 97306, France; ^2^Pediatric Unit, Cayenne Medical Center, Rue des flamboyants, BP 6006, French Guiana, Cayenne Cedex 97306, France; ^3^Faculty of Health, University of French Guiana, French Guiana, Cayenne, France; ^4^French West Indies-French Guiana Clinical Trials Center, Cayenne Medical Center, INSERM 1424, Rue des flamboyants, BP 6006, French Guiana, Cayenne Cedex 97306, France

**Keywords:** children, Cohen's d measure, depression, precariousness, sickle cell disease

## Abstract

**Importance:** While the prevalence and impact of depression have been widely described in sickle cell disease, its relationship with precariousness has never been studied.

**Objective:** This study aimed to describe the prevalence of depression and its relationship with clinical and demographic factors including social precariousness in children with sickle cell disease in French Guiana.

**Methods:** We included children aged 12–18 years with sickle cell disease from the Sickle Cell Reference Center in French Guiana. A simple depression questionnaire “Child depression inventory 2” was proposed and completed by a clinical examination and consultation by a psychologist. Using the known assessment of health inequalities and poverty in health screening centres (EPICES) score, we developed a composite precariousness score that uses five items (each item is scored from 0 to 2). According to the chosen items, precariousness was defined as a score ≥5.

**Results:** The prevalence of depression was 42.5% [95% CI: 31.5–54]. The median age was 15 years [95% CI: 13–17]. The age distribution peaked at 14 years in patients with depression. There were 76% of precarious patients in the depressed group and 18% in the control group (*p*  < 0.0001). In multivariate analysis, genotype SC (OR = 7.66, [1.17; 50.13], *p*=0.0338) and precariousness (OR = 15.68, [4.73; 51.94], *p*  < 0.0001) were associated with higher rates of depression. Baseline hemoglobin levels (OR = 0.48, [0.27; 0.88], *p*=0.0173) were also associated with lower rates of depression.

**Conclusions and Relevance:** Despite free healthcare, precariousness is an independent risk factor for depression.

## 1. Introduction

Depressive and anxiety disorders are much more common in children and adolescents with chronic illnesses [1, 2]. Their prevalence and impact have been widely described in diseases such as asthma, diabetes, and epilepsy [3–6]. Sickle cell disease (SCD) is a multisystem condition that affects more than 150 million people worldwide, primarily those of African and Mediterranean descent [7]. This disease is characterized by recurrent, acute and chronic pain, chronic anemia, and acute complications, which often require emergency management and hospitalization. Despite medical progress in the treatment of this disease, it remains a distressing condition because of the morbid and even fatal consequences that have a major impact on the quality of life of the affected patients. Depression is a mood disorder that causes persistent feelings of sadness and loss of interest [8]. The symptoms of depression in children and adolescents include sadness, irritability, clinging, worry, pain, not wanting to go to school, and being underweight. Pain, fatigue, and sleep disorders are associated with depression in children and adolescents with SCD. Many studies have been published on the prevalence and impact of depressive and anxiety disorders in SCD patients. These studies have made it possible to determine the prevalence of depression and associated risk factors [9–13]. Depression in children with SCD has been shown to increase with age and educational level [14, 15]. Some studies have shown a link between depression and family socioeconomic status (SES). Thus, the risk of depression in the family group with the lowest SES was twice as high as that in the group with the highest SES [16–18]. However, none of these studies involved children with SCD. Identifying other unknown factors associated with an increased risk of depression in children with SCD could help improve their management.

SCD is a major public health problem in French Guiana [19]. An estimated incidence of this disease at birth of 1 in 227, and the overall frequency of hemoglobin sickle cell trait (AS) carriers is 10% [19, 20]. The major groups of SCD include the three main genetic forms that combine different structural variants of hemoglobin or thalassemia syndromes (SCD-sickle cell anemia (SS), SCD-sickle hemoglobin-C disease (SC), SCD-S*β*-thalassemia (SB thal)) [21]. The affected population, predominantly of African descent, is composed primarily of three groups: Guyanese Creoles, Maroons (descendants of fugitive slaves), and, more recently, Haitian immigrants [22]. Over half of the general population lives under the poverty line in French Guiana and the communities affected by SCD are particularly concerned with poverty and often illiteracy. According to Graves, Hodge, and Jacob [10], there is a significant correlation between depression and the quality of life in children and adolescents with SCD. Their study confirmed the importance of screening for depression in children with SCD to direct them to early interventions to improve their quality of life. There are few data on the prevalence of depression in children with SCD in French Guiana. In French Guiana, healthcare is free, as in the rest of France, as long as appropriate social security coverage is available. The follow-up and management of SCD patients in French Guiana is performed from the moment of screening at birth, except for those children born outside the territory, and they are the most numerous [23]. The purpose of this study was to describe the prevalence of depression in children with SCD in French Guiana, its predictive factors, and the influence of social precariousness, a major reality in this territory.

## 2. Methods

### 2.1. Study Design

In our referral center, out of 700 patients, there were 266 children under 18 years of age, including 90 adolescents aged 12–18 years. In this cross-sectional study, we included children aged 12–18 years with SCD followed by the sickle cell reference center in French Guiana. Children included in the study were selected consecutively between January 1 and April 30, 2023, in the order of appearance based on their informed consent. A simple depression questionnaire “Children's Depression Inventory 2 (CDI 2) [24] (i)” was proposed by the therapeutic education nurse and completed by clinical examination and consultation by a psychologist. The CDI 2 is a brief self-report test that assesses cognitive, affective, and behavioral signs of depression in children and adolescents [24]. At the same time, we collected assessment of health inequalities and poverty in health screening centres (EPICES) score [25] data during the interview. Initially, we used the EPICES score in its entirety to measure vulnerability, but all the patients found it long and difficult to complete. We felt that this score was not appropriate for our study population. Based on some data from the literature [26–28], we chose criteria that were easier to understand for our study population. We therefore preferred to create a simpler score to measure precariousness, adapted from the EPICES score, which has already been validated.

### 2.2. Definition of Precariousness

The EPICES score is an individual indicator of precariousness that considers the multidimensional nature of precariousness [25]. This score has been studied and validated in chronic diseases, including type 1 diabetes in children [25]. We started by identifying predictive factors for precariousness. This led us to propose a score. We then assigned each item a maximum score of 2. We classified patients into two dichotomous categories of precariousness: low risk and high risk. As the main purpose of the score was to identify patients in a precarious situation, we chose the high-risk category so as not to miss any patients in a precarious situation. Using the EPICES score, we developed a composite precariousness score that uses the following five items (each item is scored from 0 to 2) (Table 1). According to the chosen items, precariousness was arbitrarily defined as a score ≥5. Internal validation was performed using the> bootstrapping technique, considered the most accurate by Hayes and Rockwood [29]. Our sample was randomly repeated 20 times. Twenty new samples of identical size (80 patients) were generated from the same database. Each patient could therefore be randomly included several times in one of the 20 samples, or not included at all. The score was therefore calculated on 20 different samples from the same database. This made it possible to determine the stability of the model by measuring any differences between the different samples. The score was considered stable because the replications did not show significant changes in the rankings.

### 2.3. Statistical Methods

Univariate logistic regression was performed to assess the relationship between depression and the following explanatory variables: genotype, precariousness, and SCD complications, such as hospitalization for vasoocclusive crisis (VOC), acute chest syndrome, magnetic imaging resonance abnormalities, renal failure, heart failure, cerebral vasculopathy, and biological parameters, such as baseline hemoglobin level. Using the significant variables in the univariate analysis (genotype, precariousness, and baseline hemoglobin level), we constructed a multivariate logistic regression. Data were checked for multicollinearity using the Belsley–Kuh–Welsch technique. Heteroskedasticity and normality of residuals were assessed using the Breusch–Pagan test and the Shapiro–Wilk test, respectively. Statistical significance was set at *p*  < 0.05. Statistical analyses were performed using the online application EasyMedStat (Version 3.18; www.easymedstat.com).

## 3. Results

**Figure 1:**
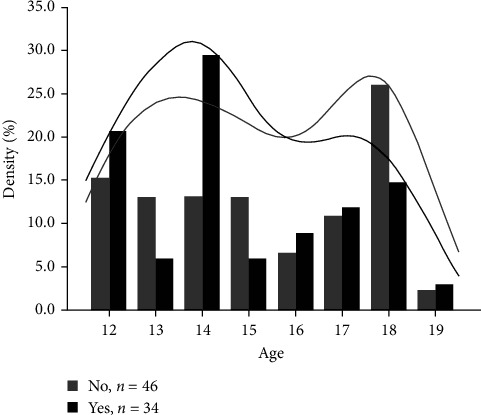
Age distribution.

Eighty patients agreed to participate in this study. The prevalence of depression was 42.5% [95% CI: 31.5–54]. There were no differences in sex or age between groups. The median age was 15 years [95% CI: 13–17]. The age distribution peaked at 14 years in patients with depression. There was no difference in age between the two groups (Figure 1). There were 76% of precarious patients in the depressed group and 18% in the control group (*p*  < 0.0001). There was no difference in baseline hemoglobin levels between the two groups.  Table 2 describes the clinical characteristics of children with SCD in relation to depression. In multivariate analysis, genotype SC (odds ratio (OR) = 7.66, [1.17; 50.13], *p*=0.0338) and precariousness (OR = 15.68, [4.73; 51.94], *p*  < 0.0001) were associated with higher rates of depression. Lower baseline hemoglobin levels (OR = 0.48, [0.27; 0.88], *p*=0.0173) were also associated with lower rates of depression (Table 3).

## 4. Discussion

Here, we show a very high prevalence of depression in a sample of children with SCD and the importance of social precariousness as a contributor. Depression is a common chronic disease [5, 6, 9, 10]. In SCD, the patient is often anxious. Indeed, repeated painful, sometimes horrible, and above all, unpredictable crises lead to a feeling of fear of the future and fear of death, fear of possible after effects [30]. Moreover, this chronic genetic disease is invisible. The only hope of a cure are bone marrow transplant or gene therapy. Studies show that these subjects are often victims of discrimination [31, 32] or incomprehension from their peers, school, and the working world [33–35]. SCD patients say that they depend on the healthcare system. As we can see in everyday practice, the healthcare system is sometimes helpless when faced with patients who experience unpredictable pain and are often hospitalized for the same symptoms with no definitive solution in sight. Depression in patients with SCD can start early in life, [23] as in our study. In addition to depression, these children also suffer from a double penalty. Families are sometimes disadvantaged and live precariously. Indeed, many young children living in poverty do not have enough to eat, as parents' circumstances make it difficult to pay rent, electricity, or grocery bills [36]. Studies have shown that over time, living with this food and social insecurity leads to delayed physical and mental development. In turn, these youths often underachieve in school, which has lifelong repercussions, leading to feelings of isolation, guilt, and stress [37]. It is not always possible to provide the essential social connections that youth need. Teens craving, independence, intimacy, and strong relationships with friends were particularly affected. Feeling embarrassed about their situation may prevent them from finding ways to express their emotions. This is true for any child but is even more remarkable for a child with SCD. Knowledge of population-specific risk factors for depression could help to ensure its prevention. In our study, although having the disease can cause depression, poor living conditions were found to be an independent risk factor. Our results are consistent with those of Lorant et al. [17]. Therefore, early identification, resulting in early intervention, has contributed substantially to improvements in psychological functioning in many chronic diseases [38]. It is likely that such improvements will also be achieved in children with SCD [39]. Indeed, community-based interventions to promote the well-being of people living with chronic diseases have a positive impact on their health status [38]. Moreover, it should be noted that perceived well-being increases with income [40, 41]. SCD often affects disadvantaged populations. Another major challenge for people with SCD is their poor access to appropriate healthcare, particularly among migrants.

French Guiana is distinguished by its high immigration rates. SCD primarily affects immigrants of Haitian origin. These immigrant populations are characterized by limited access to healthcare and more precarious living conditions. Since the economic crisis linked to the COVID-19 pandemic, hunger and poverty have reached alarming proportions in French Guiana [42], particularly among the most vulnerable populations. Although a large portion of the population is poor, French Guiana has the highest gross domestic product (GDP) per capita in Latin America, so there are great social contrasts in this small territory which may exacerbate children's awareness of their disfavored medical and social situation relative to their peers, a situation that ostracizes them at an age when “fitting in” is important.

Interestingly, there was no association between sex and depression in children in our study, whereas previous studies have shown that female sex is a significant predictor of depression in SCD [43]. Other studies have also found similar results [44, 45].

Although there was an association between SCA genotype and high rates of depression, we did not find an association between depression and disease severity. This result is consistent with the observation by previous researchers that disease severity is not a predictor of depression in SCD [46]. The association of depression with SC genotype is unexpected and could be explained by the fact that more than a third of our study population with SCD-SC are more likely to be migrants from Haiti. We found an association between depression and biological criteria such as anemia. It is noteworthy that hydroxyurea (HU) treatment did not reduce the risk of depression. This could be explained if the children were started on HU because their SCD manifestations were more severe. Regardless of its nature, suicidal ideation is common in patients with major clinical depression. The rate of suicide among children with SCD is unknown [47, 48]. According to a Jamaican study, Jamaican adolescents with SCD would have significantly higher rates of depressive symptoms [49], and the rate of suicide attempts is almost twice that of their healthy peers. However, depression may decrease adherence to care [50].

Our study has several limitations. Although we observed a high prevalence of SCD, the number of children who responded to the questionnaires was low. In addition, we were unable to study the relationship between education level and depression. Another limitation of our study is that the precariousness score may need to be validated, as the threshold of 5 was a choice. Could the results be different if a different threshold had been chosen to define precariousness? This score needs external validation.

Despite these limitations, this is the first study to demonstrate a relationship between precariousness and depression in children with SCD.

Our results suggest that healthcare providers should screen for depression and refer children with SCD for early intervention to improve their quality of life and promote good school functioning. In addition, psychological assessments should be incorporated into the routine evaluation of children with SCD during their follow-up visits, as they grow and progress through school.

## 5. Conclusion

In this study, we observed a high prevalence of depression in children with SCD. We also demonstrated that, despite free health care, precariousness was an independent risk factor for depression. Thus, to improve the quality of care, psychological assessments should be incorporated into the routine evaluation of children with SCD as they grow and progress through school. Psychological interventions for children with SCD can complement current medical treatments.

## Figures and Tables

**Table 1:** Composite precarity score.

Items/scores	0	1	2
Social security coverage	Social security/mutual/ALD*⁣*^*∗*^	AME*⁣*^*∗∗*^, CMU*⁣*^*∗∗∗*^, CMU-c	Absence of social coverage
Housing	House or apartment, each child has a room	House or apartment, shared room	Squat, no accommodation for the children
Existence of running water at home	Presence of an individual water meter	Shared meter or water collected from a neighbor	Absence of running water
Existence of electricity at home	Presence of an electricity meter	Connection from a neighbor	Absence of electricity
Net monthly income of the family	≥1500 euros	<1500 euros	Absence of formal income

Abbreviations: ALD, long-term condition; AME, state medical aid; CMU, universal health insurance; CMU-c, universal supplementary health insurance.

*⁣* ^*∗*^ALD is 100% comprehensive health coverage for chronic diseases.

*⁣* ^*∗∗*^AME is a health coverage granted to foreigners in an irregular administrative situation.

*⁣* ^*∗∗∗*^CMU and CMU-c beneficiaries are citizens whose monthly income is below the social minimum.

**Table 2:** Clinical characteristics of children with sickle cell disease in relation to depression.

Variables	Depression	No depression	OR	*p*
	*N* = 34	*N* = 46		
Age				
Mean, Std. dev	14.9 ± 2.2	15.3 ± 2.3	0.6 [0.2; 1.4]	0.4
Gender			0.4 [0.3; 2.1]	0.8
Males	14 (41%)	21 (46%)	—	—
Females	20 (59)	25 (54%)	—	—
Precarity			13.4 [4.6; 39.2]	<0.0001
Yes	26 (76%)	9 (18%)	—	—
No	8 (24%)	37 (82%)	—	—
Genotype			0.5 [0.2; 1.2]	0.2
HbSS or SB°thal	18 (53%)	32 (70%)	—	—
HbSC or SB + thal	16 (47%)	14 (30%)	—	—
History of infection			0.4 [0.1; 1.1]	0.1
Yes	23 (68%)	39 (85%)	—	—
No	11 (32%)	7 (15%)	—	—
History of ACS			0.5 [0.2; 1.3]	0.1
Yes	6 (18%)	15 (33%)	—	—
No	28 (82%)	30 (67%)	—	—
History of abnormal TCD			1.9 [0.4; 9.2]	0.4
Yes	4 (12%)	3 (7%)	—	—
No	30 (88%)	43 (93%)	—	—
History of splen acure sequestration			0.8 [0.3; 2.1]	0.7
Yes	10 (29%)	16 (36%)	—	—
No	24 (71%)	29 (64%)	—	—
History of cholelithiasis			0.5 [0.2; 1.4]	0.3
Yes	8 (24%)	17 (37%)	—	—
No	26 (76%)	29 (63%)	—	—
Treatment with HU			0.5 [0.2; 1.3]	0.2
Yes	11 (32%)	22 (48%)	—	—
No	23 (68%)	24 (52%)	—	—
Baseline hemoglobin level				
Median, IQR	8.5 [7–10]	8.5 [8–10]	2.6 [0.9; 7.3]	0.5
Baseline MCV level				
Median, IQR	75.5 [77–85]	80 [70–85]	1.8 [0.7; 4.4]	0.3

Abbreviations: ACS, acute chest syndrome; HU, hydroxyurea; IQR, interquartile range; MCV, mean corpuscular volume; Std. dev, standard deviation; TCD, transcranial doppler.

**Table 3:** Multivariate analysis of predictors of depression in children with sickle cell disease.

Variables	Depression	No depression	AOR	*p*
	*N* = 34	*N* = 46		
Age				
Mean, Std. dev	14.9 ± 2.2	15.3 ± 2.3	0.3 [0.1; 1.3]	0.1
Gender			0.5 [0.1; 2.1]	0.4
Males	14 (41%)	21 (46%)	—	—
Females	20 (59)	25 (54%)	—	—
Precarity			36.8 [6.8; 198.9]	<0.0001
Yes	26 (76%)	9 (18%)	—	—
No	8 (24%)	37 (82%)	—	—
Genotype			0.3 [0.1; 1.9]	0.2
HbSS or SB°thal	18 (53%)	32 (70%)	—	—
HbSC or SB + thal	16 (47%)	14 (30%)	—	—
History of infection			1.1 [0.2; 6.5]	0.9
Yes	23 (68%)	39 (85%)	—	—
No	11 (32%)	7 (15%)	—	—
History of ACS			0.7 [0.1; 3.9]	0.6
Yes	6 (18%)	15 (33%)	—	—
No	28 (82%)	30 (67%)	—	—
History of abnormal TCD			5.5 [0.6; 52.6]	0.1
Yes	4 (12%)	3 (7%)	—	—
No	30 (88%)	43 (93%)	—	—
History of splen acure sequestration			1.8 [0.3; 10.6]	0.5
Yes	10 (29%)	16 (36%)	—	—
No	24 (71%)	29 (64%)	—	—
History of cholelithiasis			0.8 [0.1; 7.1]	0.8
Yes	8 (24%)	17 (37%)	—	—
No	26 (76%)	29 (63%)	—	—
Treatment with HU			0.9 [0.2; 5.3]	0.8
Yes	11 (32%)	22 (48%)	—	—
No	23 (68%)	24 (52%)	—	—
Baseline hemoglobin level				
Median, IQR	8.5 [7–10]	8.5 [8–10]	13.4 [1.9; 92.8]	0.009
Baseline MCV level				
Median, IQR	75.5 [77–85]	80 [70–85]	1.2 [0.1; 2.6]	0.2

Abbreviations: ACS, acute chest syndrome; AOR, adjusted odds ratio; HU, hydroxyurea; IQR, interquartile range; MCV, mean corpuscular volume; Std. dev, standard deviation; TCD, transcranial doppler.

## Data Availability

The data will be made available upon request.
